# The extract from *Agkistrodon halys* venom protects against lipopolysaccharide (LPS)-induced myocardial injury

**DOI:** 10.1186/s12906-019-2595-4

**Published:** 2019-07-17

**Authors:** Quan-Hai Wang, Wei Li, Yu-Xin Jiang, Xiao-Hua Lu, Guo-Guang Wang

**Affiliations:** 1 Department of Histology and Embryology, Wannan Medical College, Wuhu, 241002 People’s Republic of China; 2 Department of Pathophysiology, Wannan Medical College, 22 West Wenchang Road, Yijiang District, Wuhu, 241002 China; 3 Department of Physiology, Wannan Medical College, Wuhu, 241002 People’s Republic of China

**Keywords:** *Agkistrodon halys* venom, LPS, Myocardial injury

## Abstract

**Background:**

Snake venoms contain various bioactive constituents which possess potential therapeutic effects. The aim of this work was to investigate the effect of the extract from *Agkistrodon halys* venom on lipopolysaccharide (LPS)-induced myocardial injury.

**Methods:**

Thirty male Sprague-Dawley rats were randomly assigned to three groups (10 rats per group): control group, LPS group and LPS + extract group. Rats in control and the LPS groups were intravenously injected with sterile saline solution, and rats in the LPS + extract group with the extract. After 2 h, rats of the control group were intraperitoneally injected sterile saline solution, and rats in the LPS and the LPS + extract groups were treated with LPS (20 mg per kg body weight). Levels of creatine kinase (CK) and lactate dehydrogenase (LDH) in serum were determined. Anti-inflammation of the extract was analyzed via determination of TNF-α and IL-6 in serum, and expression of TNF-α, IL-6, COX-2 and p-ERK protein in hearts. Heme oxygenase-1 (HO-1) and p-NF-κB protein expression in hearts, superoxide dismutase (SOD) activity and malondialdehyde (MDA) level in serum were used to evaluate the anti-oxidative properties of the extract.

**Results:**

Extract pretreatment significantly decreased the level of serum CK and LDH, reduced the generation of inflammatory cytokines such as TNF-α and IL-6, and also reduced serum level of MDA in the LPS + extract group compared with the LPS group. In addition, the extract increased SOD activity in serum, HO-1 protein expression in hearts, and decreased TNF-α, IL-6, COX-2, p-NF-κB and p-ERK1/2 protein expression.

**Conclusion:**

Our results suggested that beneficial effect of this extract might be associated with an improved anti-oxidation and anti-inflammatory effect via downregulation of NF-κB/COX-2 signaling by activating HO-1/CO in hearts.

## Background

Acute myocardial infarction, same as sepsis, is a major cause of high mortality [1]. Lipopolysaccharide (LPS), a bacterial endotoxin located in the outer membrane of the cell wall of the Gram-negative bacteria, is considered as the principal factor responsible for multiple organs damage including myocardial injury in patients with sepsis [2, 3]. Indeed, various evidences show that myocardial injury is the principal contributor to mortality in septic patients [4, 5]. LPS results in the overexpression and release of proinflammatory cytokines, including tumor necrosis factor-α (TNF-α), interleukin-1β (IL-1β), and interleukin-6 (IL-6), which contribute to LPS-induced multiple organs failure including myocardial depression [6, 7]. Furthermore, LPS activates nuclear factor-κB (NF-κB) and mitogen-activated protein kinase (MAPK) pathways via recognizing Toll-like receptors (TLR), which upregulate the expression of cyclooxygenase-2 (COX-2), subsequently promoting the synthesis and release of inflammatory cytokines [8, 9]. In addition, LPS increases reactive oxygen species (ROS) generation, oxidative stress and activation of stress signaling including mitogen-activated protein kinase (MAPK), which promote apoptosis of cardiac myocytes and myocardial dysfunction in sepsis [10, 11]. LPS is a crucial factor generating multi-organ failure in sepsis, including cardiac failure confirmed by experimental and clinical studies [12–14]. Accordingly, inhibition of proinflammatory cytokines is beneficial to reduce septic shock [15, 16], and antioxidants attenuate LPS-induced myocardial depression [17, 18].

Snake bite is dangerous to public health due to its hemotoxic, neurotoxic, and cardiotoxic effects, which can result in death. However, snake venoms contain various bioactive constituents which possess potential therapeutic effects [19]. Snake venoms are complex mixtures composed of proteins, polypeptides and non-protein components such as metalloproteases and phospholipase A2 [20]. Many researchers pay great attention to the medical value of snake venom components. Many progresses have been made in the use of snake venom constituents to develop medications including anti-hypertensive and anti-stroke drugs, and some studies suggested that venom extracts possess anti-cancer activity [21, 22]. Several proteins separated from snake venoms affect blood circulation and coagulation, for example, the extract from *Agkistrodon contortrix* venom can activate protein C (PC) [23, 24]. Activated PC (APC) results from the circulating PC cleaved by thrombin and it plays a vital role in coagulation homeostasis. Furthermore, APC increases the survival of patients with sepsis through its anti-inflammatory effect [25] by reducing both NF-κB pathway and expression of pro-inflammatory cytokines such as TNF-α and IL-6 induced by LPS [26, 27]. Thus, PC activator (PCA) might increase the anti-inflammatory effect. Recently, we isolated a component with enzymatic activity from *Agkistrodon halys’* venom. An experimental study indicated that it can activate PC. We also investigated the functions of the extract, and the results showed that it improves cardiac hemodynamics in septic shock rats. Furthermore, according to the literature, this extract alleviates diabetic cardiomyopathy, and increases anti-inflammation in STZ-induced diabetic rats [28]. In addition, we also separated other components with bioactivity from snake venoms. Preliminary studies suggested that some peptides displayed analgesia, and the protein could attenuate cerebral ischemia/reperfusion injury, but these components need further identification. In this study, we hypothesize that PCA from snake venom attenuates LPS-induced myocardial injury on the basis of previous results. Therefore, in the present work the effect of the extract on myocardial injury in sepsis induced by LPS was further analyzed, together with its anti-inflammatory and antioxidative effects.

## Methods

### Materials

Lipopolysaccharide (LPS, *Escherichia coli* 0111:B4) was purchased from Sigma (St. Louis, USA). ELISA rat IL-6 and TNF-α kits were purchased from Hefei Bomei Biotechnology CO., LTD (Hefei China). Primary polyclonal antibodies β-actin, HO-1, COX-2, NF-κB, p-NF-Κb, p-ERK1/2, ERK were purchased from Bio Basic Inc., Canada.

### Animals

Male Sprague-Dawley rats (260-300 g) were provided by the Experimental Animal Center in Wannan Medical College (Wuhu, China) and raised in the animal house facilities of the Experimental Center for Function Subjects at Wannan Medical College under a 12-h day/light cycle and controlled temperature of 22 °C. Rats were fed with a standard pellet diet and they had access to water ad libitum. This project was approved by the Academic Experimental Animal Care and Use Committee of Wannan Medical College.

### Extract of *Agkistrodon halys* venom

The extract from *Agkistrodon halys* venom was supplied by the Snake Venom Research Institute, Wannan Medical College. The extract used in the study displays high similarity with acurhagin precursor with activity of metalloproteinase. The lyophilized extract was stored at − 20 °C. Extract solution (1 mg/ml) was prepared in sterile saline solution. Number: 20130041502.

### Induction of myocardial injury

A total of thirty male Sprague-Dawley rats were used in this work. After acclimatization for 1 week, rats were randomly assigned to three groups (10 rats per group): control group, LPS group and LPS + extract group. Rats were anesthetized by an intraperitoneal injection of sodium pentobarbital (40 mg/kg, i.p.) (Sigma, St. Louis, USA). Rats of the control and LPS groups were intravenously treated with sterile saline solution, and rats of the LPS + extract with the extract from snake venom (100 μg/kg). Myocardial injury was induced as described previously [9]. Briefly, after 2 h of injection with the extract, rats from the LPS and LPS + extract groups were intraperitoneally injected with LPS (20 mg per kg body weight, dissolved in sterile saline) (Sigma, St. Louis, USA) for cardiomyopathy induction, while the control group were treated again with sterile saline solution (intraperitoneally) as control. After 6 h of LPS administration, the carotid artery of rat was seperated and cannulated for collection of blood sample. Further, animals were anaesthetised via bleeding. Blood samples were collected for biochemical analysis. Hearts were removed for each group, and six of them were stored at − 70 °C until use and the other four were fixed in 4% neutral formalin for histological analysis. At the end of experiment, rats’ survival rate was 10/10 in each group.

### Biochemical analysis

Blood samples were centrifuged to obtain the serum. Serum levels of creatine kinase (CK) and lactate dehydrogenase (LDH) were determined by an automated biochemical analyzer.

### Analysis of antioxidation

To evaluate effect of the extract on the antioxidation in the serum, superoxide dismutase (SOD) activity was measured by the xanthine oxidase method, and malondialdehyde (MDA) content was determined by the thiobarbituricacid method using diagnostic kits (Nanjing Jiancheng Bioengineering Institute, Nanjing, China) according to the manufacturer’s protocol. Serum diluted with 10 mmol/L NH_2_OH•HCl, 7.5 mmol/L xanthine and 0.2 mg/ml xanthine oxidase was incubated at 37 °C for 30 min, and then was mixed with chromogenic agent for 10 min. Absorbance at 530 nm was determined for measurement of SOD activity. Mixture of serum with 0.6% thiobarbituricacid (1:1, v/v) was boiled for 15 min, and then centrifuged for 10 min. MDA content was measured by determining absorbance at 532 and 450 nm.

### Determination of TNF-α and IL-6

TNF-α, and IL-6 contents in serum were evaluated using TNF-α, and IL-6 specific ELISA kits (Hefei Bomei Biotechnology CO., LTD, China) according to the manufacturer’s instructions.

### Morphometric analysis

Hearts fixed in 4% neutral formalin were embedded in paraffin after dehydration, and cut into 5-μm sections used for hematoxylin-eosin staining. Stained sections were observed under a light microscope for histomorphological analysis.

### Western blot

Hearts stored at − 70 °C were lysed and homogenized inice-cold lysis buffer (50 mmol/L HEPES, 100 mmol/L sodium pyrophosphate, 10 mmol/L sodium orthovanadate, 100 mmol/L sodium fluoride, 10 mmol/L EDTA, and 1% Triton X-100) containing 2 mmol/L phenylmethyl sulfonyl fluoride. Homogenates were centrifuged at 12,000 g for 20 min at 4 °C. Equal amounts of denatured protein in supernatants were separated by electrophoresis on 12% SDS-PAGE and then transferred onto nitrocellulose membranes. The membranes were incubated with rabbit anti-rat primary antibodies such as β-actin, HO-1, COX-2, NF-κB, p-NF-κB, p-ERK1/2, ERK, (Bio Basic Inc., Canada) overnight at 4 °C. Subsequently, membranes were incubated with a horseradish peroxidase-conjugated secondary goat anti-rabbit antibody. After rinsing, proteins were visualized by DAB staining (Bio Basic Inc., Canada).

### Statistical analysis

Statistical analysis was performed using SPSS16.0 (IBM Corp., Armonk, NY, USA). Tukey’s test for unpaired data and one-way Analysis of Variance (ANOVA) were used to determine statistical differences, followed by Bonferroni’s post-test. All values were expressed as mean ± SD. A *p* value < 0.05 was considered statistically significant.

## Results

### Effect of the extract on CK and LDH

The extract used in the study was a component which we recently isolated from *Agkistrodon halys* venom. The extract has been identified, and its function has been reported in previous study [28].

**Fig. 1:**
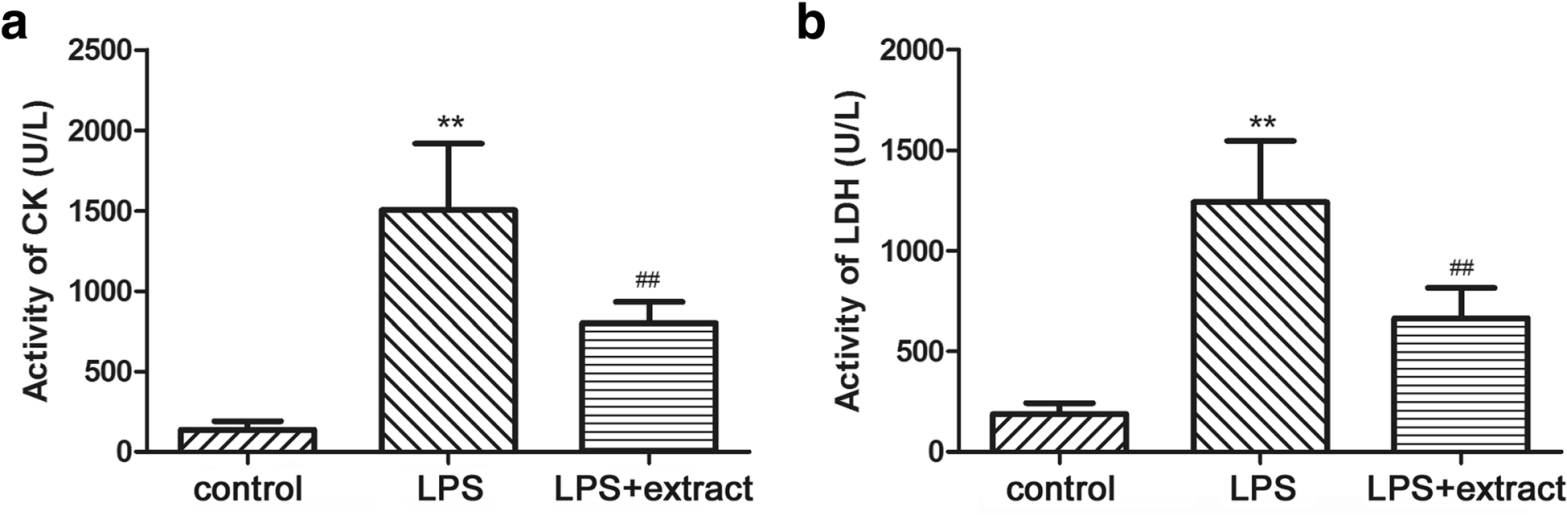
Effect of venom extract on CK and LDH in rat serum. Activity of CK (**a**) and LDH (**b**) was determined by automated biochemical analyzer. The results were expressed as U/L in serum. Values were expressed as means ± SD. **p < 0.01 vs. Control group; ^##^*p* < 0.01 vs. LPS group (*n* = 10)

To evaluate effect of the extract on LPS-induced myocardial injury, CK and LDH serum levels were determined. As shown Fig. 1, CK and LDH levels were significantly increased in LPS group when compared to the control (*p* < 0.01), while the extract treatment markedly reduced their levels in the LPS + extract group compared to the LPS group (*p* < 0.01).

### Change of antioxidation

**Fig. 2:**
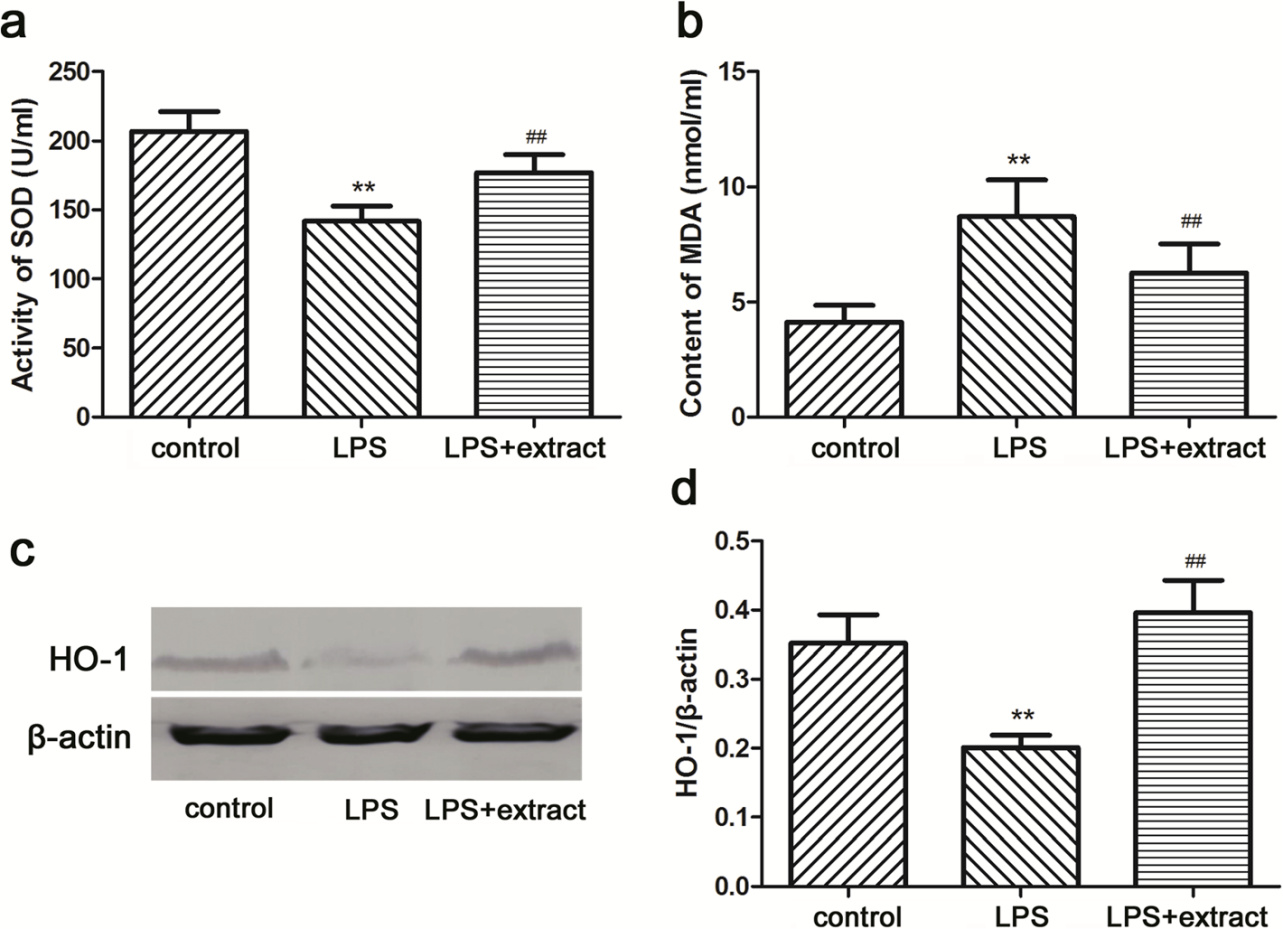
Effect of the venom extract on antioxidants. Serum SOD activity (**a**) and MDA content (**b**) were measured. The results were expressed as means ± SD (*n* = 10). HO-1 expression in hearts was analyzed by western blot (**c**). Relative amount of HO-1 was assessed by HO-1 to β-actin ratio (**d**) (*n* = 6). **p < 0.01 vs. Control group; ^##^*p* < 0.01 vs. LPS group

LPS treatment significantly reduced SOD activity, and increased the content of MDA in serum from rats of the LPS group compared with the control group (*p* < 0.01) (Fig. 2). Extract administration enhanced SOD activity, and decreased MDA content when compared to the LPS + extract group (*p* < 0.01) (Fig. 2).

Furthermore, HO-1 expression was detected in myocardium. The data showed that its expression was decreased in the LPS group compared with the control group (*p* < 0.01) (Fig. 2), while the extract significantly increased HO-1 expression in LPS + extract group when compared to the LPS group (*p* < 0.01) (Fig. 2).

### Anti-inflammatory effect of the extract

**Fig. 3:**
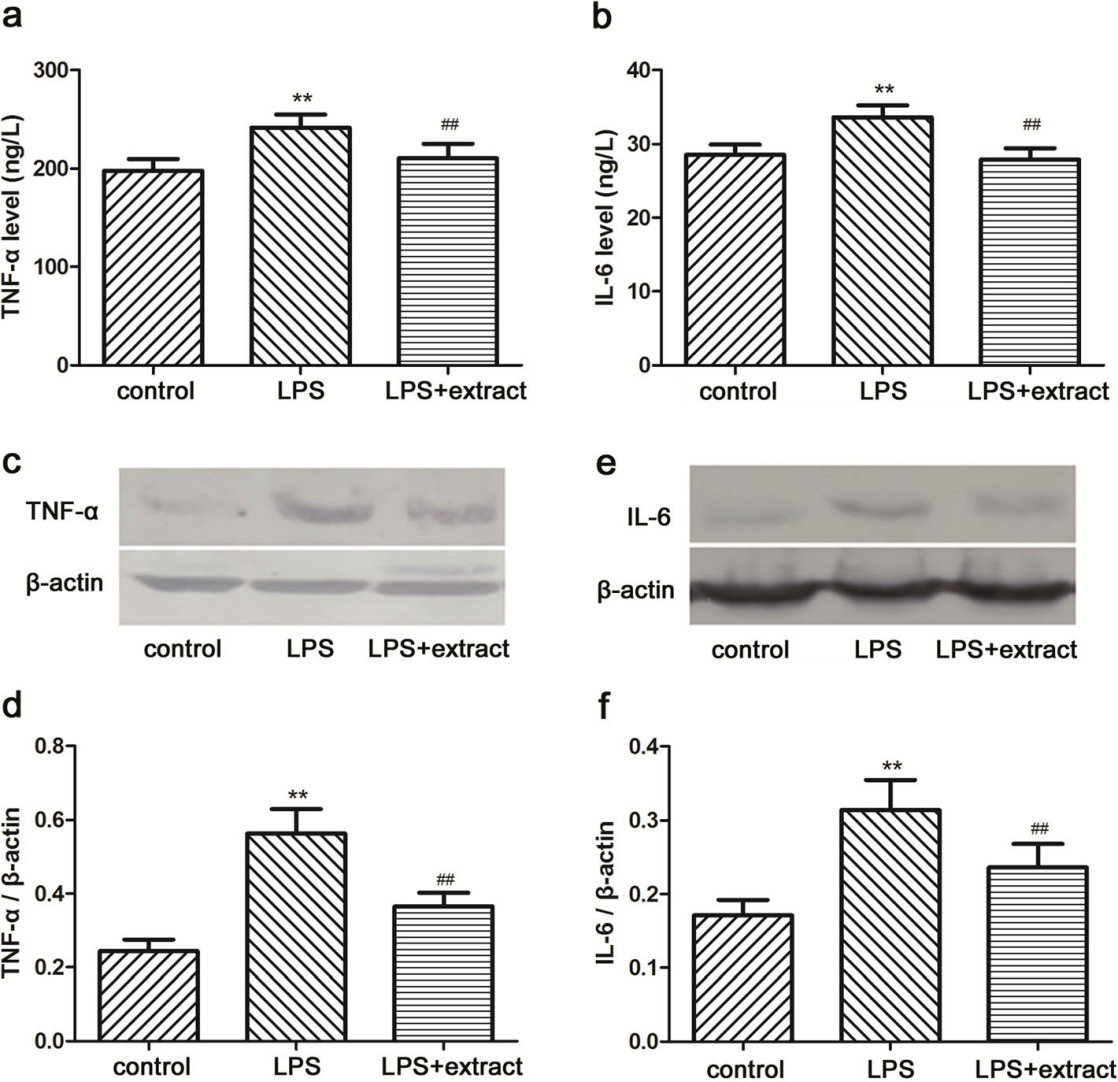
Effect of the venom extract on inflammation. Seruml evels of TNF-α (**a**) and IL-6 (**b**) were determined by TNF-α, and IL-6 specific ELISA kit. Values were expressed as means ± SD (*n* = 10). TNF-α (**c**) and IL-6 (**e**) in hearts were analyzed by western blot. Relative amounts of TNF-α (**d**) and IL-6 (**f**) were assessed by TNF-α and IL-6 to β-actin ratio (*n* = 6), respectively. **p < 0.01 vs. Control group; ^##^*p* < 0.01 vs. LPS group

Levels of TNF-α and IL-6 in serum were significantly increased in the LPS group compared with the control group (*p* < 0.01) (Fig. 3). Treatment with extract prior to LPS markedly decreased TNF-α and IL-6 levels compared to the LPS group (*p* < 0.01) (Fig. 3).

In addition, LPS treatment significantly increased TNF-α and IL-6 levels in the heart of rats from LPS group compared with the control group (*p* < 0.01) (Fig. 3), while they were reduced in LPS + extract group (*p* < 0.01) (Fig. 3).

### Morphometric change

**Fig. 4:**
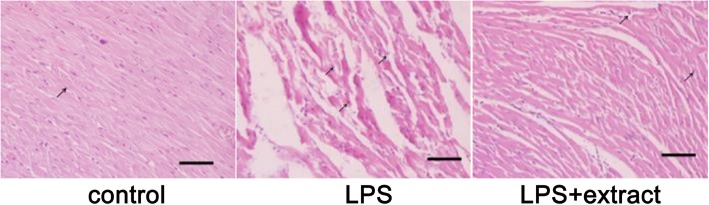
Morphometric change. Heart samples were collected 6 h after injection with LPS, fixed in formalin, and sections were cut and stained with hematoxylin-eosin. Scale bar: 50 μm. 400 × magnification

Histological observation showed that inflammatory cell infiltration was increased in the hearts of the LPS group compared with the ones from the control group. Myofibrillar fragmentation and cardiacmyocytes swelling were found in the LPS group (Fig. 4). Treatment with the extract reduced both these effects induced by LPS (Fig. 4).

### COX-2, p-ERK1/2, and p-NF-κB protein expression

**Fig. 5:**
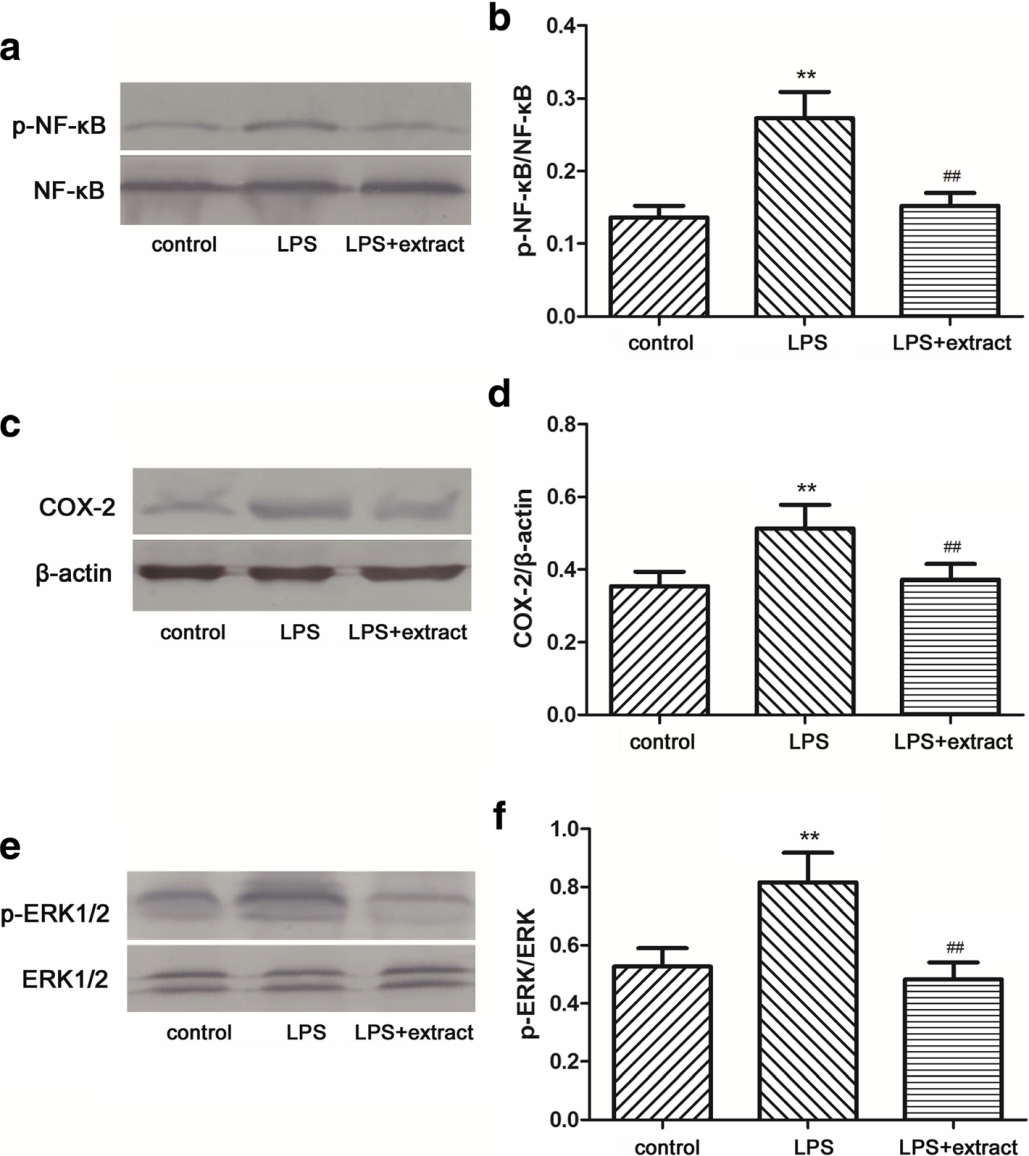
p-NF-κB, COX-2, and p-ERK1/2 protein expression in hearts. p-NF-κB(**a**), COX-2(**c**), and p-ERK1/2 (**e**) protein expression in hearts was analyzed by western blot. Relative amounts of p-NF-κB, COX-2, and p-ERK1/2 were assessed by p-NF-κB to NF-κB(**b**), COX-2 to β-actin(**d**) and p-ERK1/2 to ERK1/2 (**f**) ratio, respectively. **p < 0.01 vs. Control group; ^##^*p* < 0.01 vs. LPS group (*n* = 6)

p-NF-κB, COX-2, and p-ERK protein expression was measured to evaluate inflammation. As shown in Fig. 5, exposure to LPS increased the expression of NF-κB, COX-2, and p-ERK proteins in heart from in the LPS group when compared to the control group. Pretreatment with the extract reduced the expression of all the mentioned proteins.

## Discussion

In the present study, we explored the protective effect of the extract from *Agkistrodon halys* venom on LPS-induced myocardial injury. Our results showed that this extract significantly decreased plasma CK and LDH level, lowered the serum level of pro-inflammatory cytokines such as TNF-α and IL-6, and the content of lipid peroxidation product such as MDA. Pretreatment with the extract also enhanced serum SOD activity, increased HO-1 expression, but reduced p-NF-κB, COX-2, and p-ERK expression in rat hearts. These results suggest that this extract was beneficial to LPS-induced myocardial injury.

Oxidative stress and inflammatory response play an important role in acute and chronic myocardial injury [29]. Inflammatory cytokines such as TNF-α and IL-6 result in cardiac dysfunction in sepsis [30]. LPS dramatically stimulates the release of inflammatory cytokines including TNF-α via binding to toll-like receptor-4 (TLR-4) [31]. Clinical and experimental studies indicated that cardiac dysfunction is closely associated with the release of inflammatory cytokines in sepsis [12, 32], and cardiac function can be improved via decreasing TNF-α level in septic animal model and patients with sepsis [33, 34]. Oxidative stress results in excessive generation of ROS and MDA, and reduces activity of anti-oxidative enzymes such as SOD, and HO-1 protein expression [35]. Excessive ROS caused damage to cells and various tissues through impairing macromolecules such as protein and DNA, and mitochondrial function [36]. Accumulating evidence shows that inflammation provokes oxidative stress, and oxidative stress enhances inflammation [37]. APC decreases the level of LPS-induced pro-inflammatory cytokines in sepsis [27]. In the present study, the extract from *Agkistrodon halys* venom, an activator of protein C, was used to pretreat rats before LPS injection. The results showed that the extract decreased the level of inflammatory cytokines such as TNF-α and IL-6 levels, and MDA content in serum. The extract also increased SOD activity.

A previous study showed that the extract can activate PC [28]. APC has been reported to possess anti-inflammation and anti-apoptotic effect [38]. Thus, we preliminarily investigated the effect of the extract on inflammation. LPS can upregulate mitogen-activated protein kinase (MAPK)/extracellular signal-regulated kinase (ERK), and enhance inflammatory response [39, 40]. Furthermore, reduction of ERK activation attenuates cardiac fibrosis [41]. In addition, LPS binding to TLR4 results in inflammation, which is associated with NK-κB activation [42]. Phosphorylation of NK-κB results in the release of NK-κB bound to the inhibitor IκB [43, 44]. Dimerized NK-κB moves to nucleus, and stimulates the expression of genes associated with inflammation including inflammatory cytokines, and COX-2 [45]. Furthermore, ERK1/2 and MAPK are also implicated in the regulation of inflammatory cytokines and COX-2 [46, 47]. In our study, the results suggested that the extract reduced inflammatory cytokines, COX-2 protein expression, and activation of ERK1/2, NK-κB and MAPK. Therefore, the protective effect of the extract might be associated with its anti-inflammatory effect.

In this study, pretreatment with the extract increased HO-1 expression in the heart. HO-1, an endogenous antioxidant, alleviates oxidative stress, and protects cells and tissues from the effect of oxidative stress [48, 49]. HO-1 can convert heme into carbon monoxide (CO), biliverdin, and free iron. Various studies showed that the conversion products such as biliverdin and CO possess antioxidative effects [50, 51]. CO, a catalytic product of HO-1, is associated with diverse physiological action [52]. CO, regarded as a gas signal molecule, exerts antioxidative, anti-inflammatory and anti-apoptotic effects [53]. Previous studies suggested that HO/CO pathway is involved in mediation of inflammation and cytoprotection [54, 55]. This study indicated that HO-1 could reduce the production of inflammatory cytokines, the expression of COX-2, and MDA generation [56].

## Conclusions

In conclusion, our study showed that the extract from *Agkistrodon halys* venom alleviated LPS-induced myocardial injury. Its beneficial effect might be associated with its effect on improvement of antioxidation and reduction of inflammation by increasing HO-1 expression, and downregulating p-NF-κB/COX-2 signaling in hearts.

## Data Availability

The datasets used and analysed during the current study are available from the corresponding author on reasonable request.

## Abbreviations

CK: Creatine kinase
CO: Carbon monoxide
COX-2: Cyclooxygenase-2
ERK: Extracellular signal-regulated kinase
HO-1: Heme Oxygenase-1
IL-6: Interleukin-6
LDH: Lactate dehydrogenase
LPS: Lipopolysaccharide
MAPK: Mitogen-activated protein kinase
MDA: Malondialdehyde
NF-κB: Nuclear factor kappa B
PCA: Protein C activator
ROS: Reactive oxygen species
SOD: Superoxide dismutase
TLR: Toll-like receptors
TNF-α: Tumor necrosis factor-α
